# Alcohol as friend or foe in autoimmune diseases: a role for gut microbiome?

**DOI:** 10.1080/19490976.2021.1916278

**Published:** 2021-07-05

**Authors:** Blaine Caslin, Kailey Mohler, Shreya Thiagarajan, Esther Melamed

**Affiliations:** Department of Neurology, Dell Medical School, The University of Texas at Austin, Austin, United States

**Keywords:** Alcohol, autoimmunity, microbiome, inflammation, dysbiosis

## Abstract

Alcohol is well known for promoting systemic inflammation and aggravating multiple chronic health conditions. Thus, alcohol may also be expected to serve as a risk factor in autoimmune diseases. However, emerging data from human and animal studies suggest that alcohol may in fact be protective in autoimmune diseases. These studies point toward alcohol’s complex dose-dependent relationship in autoimmune diseases as well as potential modulation by duration and type of alcohol consumption, cultural background and sex. In this review, we will explore alcohol’s pro- and anti-inflammatory properties in human and animal autoimmune diseases, including autoimmune diabetes, thyroid disease, systemic lupus erythematosus, rheumatoid arthritis, experimental autoimmune encephalomyelitis and multiple sclerosis. We will also discuss potential mechanisms of alcohol’s anti-inflammatory effects mediated by the gut microbiome.

## Introduction

Autoimmune diseases arise from aberrant immune system activation against self-antigens and affect approximately 24 million people in the United States, with rising incidence in recent years.^1^ Women tend to be disproportionately affected, with female-to-male odds ratios of up to 9:1 in some autoimmune diseases.^2^ While there are known genetic risk alleles,^3^ environmental factors are increasingly seen as major contributors to triggering autoimmunity.^4^ Among environmental factors, diet and the composition of the gut microbiome are being closely studied for their role in the initiation and progression of autoimmune disorders.^5,6^

Alcohol is a widely available dietary factor in our society and its pro-inflammatory effects and end-organ damage are well documented at high doses.^7^ However, alcohol’s role in inflammation and autoimmunity at moderate doses has been relatively less well understood. While it may be hypothesized that alcohol could serve as an environmental inflammatory risk factor, recent evidence actually points toward alcohol’s protective effects in several autoimmune diseases, including autoimmune thyroid disease, autoimmune diabetes, systemic lupus erythematosus (SLE), rheumatoid arthritis (RA) and multiple sclerosis (MS), both in human and animal studies.^8–15^ Although it is perplexing to explain alcohol’s apparent protective role in autoimmune diseases given its pro-inflammatory properties, current evidence suggests that alcohol has pleiotropic tissue-specific and sex-specific anti-inflammatory actions in the body at different doses.

In this review, we will examine alcohol’s dose-dependent effects and potential mechanisms in autoimmune diseases in human and animal studies with a focus on the role of alcohol in modulating the gut microbiome in autoimmunity.

## Alcohol metabolism and dosing in human and animal studies

**Figure 1.**
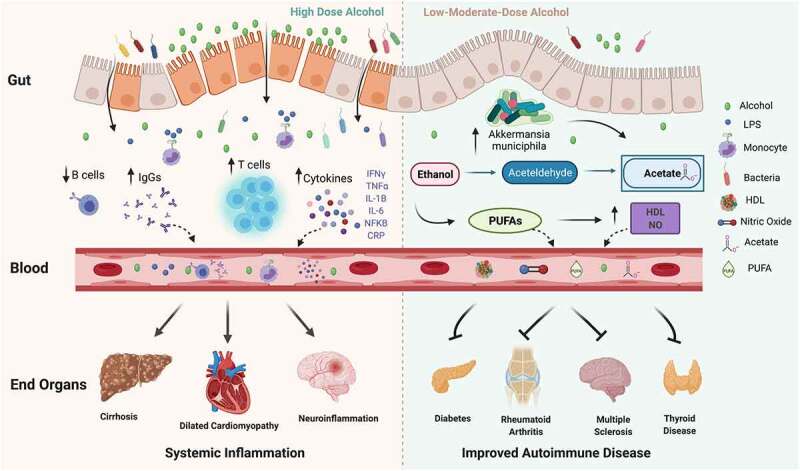
Alcohol has pleiotropic effects in the body. At high doses, alcohol destabilizes the gut barrier and can lead to dysbiosis, increase in bacterial wall product, liposaccharide (LPS), which can stimulate toll-like receptors (TLR) on immune cells and lead to an increase in monocytes, T cells, cytokines and immunoglobulin (IgG) levels as well as a decrease in B cells. In turn, circulating inflammatory cytokines, IgGs and immune cells contribute to end-organ damage. At low-moderate doses, alcohol has been demonstrated to improve autoimmune disease risk and progression. Although the precise mechanism is not well understood, low-moderate alcohol may have a positive impact on inflammation via increase in *Akkermansia muciniphila* and other protective gut microbes, as well as contribute to increases in acetate, polyunsaturated fatty acids (PUFAs), high-density lipoprotein (HDL) and nitric oxide (NO)

Shortly after consumption, alcohol is absorbed into the bloodstream from the stomach and small intestine, and subsequently diffuses to different body organs. Alcohol is metabolized to acetaldehyde and acetate primarily within the gastrointestinal (GI) tract, but also in other organs, such as the brain.^16^ Both alcohol and acetaldehyde can induce systemic inflammation via 1) activation of toll-like receptors (TLRs) 2, 3, 4 and NOD-like receptor family pyrin containing 3 (NLRP3) inflammasome complex on immune cells, 2) bacterial overgrowth in the GI tract and production of a bacterial breakdown product, lipopolysaccharide (LPS) and 3) generation of free oxygen radical species and inducible nitric oxide synthase (iNOS), both of which directly affect the permeability of gut tight junctions leading to LPS leakage into the bloodstream^17–20.^ LPS is a strong TLR agonist and leads to the activation and maturation of macrophages and other innate immune cells.^21^ Thus, the combination of increased gut permeability, LPS translocation and alcohol-mediated immune activation can predispose to a pro-inflammatory state (Figure 1).

In interpreting human and animal alcohol studies, it is important to closely consider the administered quantity of alcohol. Patterns of human drinking are typically divided into light, moderate and heavy consumption. For humans, a standard alcoholic drink is defined as approximately 14 g of alcohol.^22^ According to the CDC, light drinking is considered to be three or fewer alcoholic drinks per week. Moderate drinking is defined as one alcoholic drink per day for women and two drinks per day for men, though variations across studies exist for this definition. Heavy alcohol consumption is defined as having four or more drinks/day for females and five or more drinks/day for males.^22^ Accurate human consumption can be challenging to quantify due to participant subjective memory and accurate reporting.

In animal studies, it is also important to account for variable physiological effects due to administered dose, route of administration (e.g. oral vs. gavage vs. intravenous), consumption pattern (e.g. voluntary vs. non-voluntary), as well as differences in body weight and metabolism between animal species. Rodents, for example, metabolize alcohol approximately five times faster than humans, which results in relatively lower voluntary consumption as rodents rapidly experience the deleterious effects of acetaldehyde accumulation.^23^ In animal studies, alcohol consumption is usually measured in grams of pure ethanol per kilogram of body weight (g/kg), though some studies may report values as blood ethanol concentration (BEC) or as alcohol by volume (ABV).^24^ For murine alcohol studies, light, moderate and heavy alcohol consumption are generally within 0–1.5 g/kg, 2.5 g/kg and 3–6 g/kg, respectively.^25–27^

## Protective role of light-moderate dose of alcohol in autoimmune diseases

Alcohol consumption may be expected to contribute toward an increased risk of or exacerbation of autoimmune diseases given its pro-inflammatory properties. Indeed, in some inflammatory diseases, such as irritable bowel syndrome (IBS) and perennial allergies, there is a direct correlation between consumption of high alcohol doses and disease onset.^28,29^ However, impressively, in multiple studies across autoimmune diseases, light-moderate alcohol consumption appears to reduce the disease risk, severity and progression (Table 1). In the following section, we will delineate the known alcohol dose-dependent effects on autoimmune diseases.

**Table 1.** Alcohol’s therapeutic effects in autoimmune diseases

Disease	Study Design	No. cases/controls	Evaluated Alcohol Dose(s)	Study Duration/Region	Impact of Light-Moderate Alcohol on Disease Risk/Morbidity	Reference
Systemic Lupus Erythematosus	Case Control	150/300	Light, Moderate, Binge (0–10 units of alcohol)	1993–1995 Nottingham, UK	↓	Hardy CJ et al, 1998
	Case Control	85/205	Moderate (1–150 g/month, >150 g/month)	1981–1999 Sweden	↓	Bengtsson AA et al, 2002
	Meta Analysis	822/55,363 6 case control studies	Moderate	Japan, Sweden, US, UK	↓	Wang J et al 2008
	Cohort	127/64,500	Moderate (wine >4 drinks/week)	1995–2015 US Black Women’s Health Study	↓	Cozier YC et al 2019
	Cohort	67/57,613	Light, Moderate (<1; 1–6; >7 drinks/week)	1997–1999 US Black Women’s Health Study	No effect	Formica MK et al, 2003
	Cross-sectional	505	Light drinkers (1–5; >6 glasses/week)	2014–2016 Korean Lupus Network	No effect	Kim SK et al, 2017
	Cross-sectional	244/204,055	Light to Moderate (0–5 g/d or >5 g/d)	1988–1990 Nurse Health Study 1 and 1996–1999 Nurse Health Study 2	↓	Hahn J et al, 2020, Barbhaiya M et al, 2017
Rheumatoid Arthritis	Cross-sectional	2800/2102	Hazardous vs. non-hazardous (1–2 drinks, 3–4 drinks, 5–6 drinks, 7–9 drinks, >10 drinks)	1992–2005 Sweden; Better Anti- Rheumatic FarmacOTherapy	↓	Bergman S et al, 2013
	Cross-sectional	3353/2836	Low, Moderate (0–16 drinks/week)	1996–2014 Sweden	↓	Hedstrom AK et al, 2019
	Cohort	11,839	Mild (≤7 units per week); Moderate (8–14 unites/week); Moderate-High (15–21 units/week); High (>21 units/week)	1987 and 2016 UK Clinical Practice Research Datalink	↓	Humphreys JH et al, 2017
	Meta Analysis	1878/195,029 8 prospective studies	Light to Moderate	Studies completed prior to 2013	J shaped	Jin Z et al, 2014
	Cross-sectional	188	Light to Moderate (nondrinkers, participants who consume 1–7 drinks/week, 8–14 drinks/week and >14 drinks/week)	2013–2014 Leiden Early Arthritis Clinic	J shaped	Mangnus L et al, 2018
	Cohort	596	Moderate abstinent (0 g/d), moderate (≤20 g/d for women, ≤30 g/d for men), and abuse (>20 g/d for women, >30 g/d for men)	2002–2005 France Étude et Suivi des Polyarthrites Indifférenciées Récentes	↓ in males, ↑ in females	Sageloli F et al, 2018
	Cohort	166	Moderate <15 or >15 beverages per month	2002–2005 Consortium for the Longitudinal Evaluations of African Americans with Early Rheumatoid Arthritis (CLEAR) registry1	J shaped	Davis ML et al, 2013
	Cross-sectional	174/204,055	Moderate (0–25 g/d)	1988–1990 Nurse Health Study 1 and 1996–1999 Nurse Health Study 2	U shaped	Lu B et al, 2010
	Cohort	662/1100	Moderate (5.1–10.0 g/d)	2003–2010 Brigham and Women’s Hospital Rheumatoid Arthritis Sequential Study	J shaped	Lu B et al, 2014
	Animal Research	5–10/group	Moderate Consumption (10% v/v ethanol)	42 d	↓	Azizov V et al, 2020
	Animal Research	12/group	Moderate alcohol (10% ethanol in water)	6 weeks	↓	Jonsson IM et al, 2007
Thyroid Disease	Case Control	140/560	Low (1–10 units per week), moderate (11–20 units per week), high (>21 units/week)	1997–2001 Denmark	↓	Carle A et al, 2012
	Case Control	272/1088	Light, Moderate and hight (0, 3–10, 11–20 or ≥21 units/week)	1997–2001 Denmark	↓	Carle A et al, 2013
	Case Control	803/1003	<10 or >10 units/week	5 year study Amsterdam Autoimmune thyroid disease	↓	Effraimidis G et al, 2012
	Case Control	140/5154(hyperthyroidism) 206/5154 (hypothyroidism)	35 g/d	2007–2009 Chinese She population	↓	Huang Y et al 2019
Diabetes	Cohort	186/2781	HUNT 1: abstainers, <1, 1–4, 5–10 and >10 times and HUNT 2: abstainers, very low (0.01–4.9 g/d), low (5–9.9 g/d), moderate (10–14.9 g/d) and high consumers (>15 g/d)	1984–2008 Norway Nord-Trondelag Health Survey (HUNT) studies	↓	Rasouli B et al, 2013
	Meta-analysis	13 cohorts	Moderate (5–30 g alcohol/d)	Caucasians, Japanese, African Americans, Nauruans	↓	Carlsson S et al, 2005
	Case Control	1905/51,529	Light (0–4.9 g/d) vs. Moderate (5.0–29.9 g/d)	1986–2004 Health Professionals Follow-Up Study	↓	Joosten MM et al, 2011
	Cohort	8289	0, low (less than or equal to 14/7 (men/women) drinks/week), risky (more than 14/7 drinks and less than 28/14 drinks/week), and high (more than 28/14 drinks/week)	1979–2012 US National Longitudinal Survey of Youth	↓	Kerr WC et al, 2018
	Meta-analysis	26 studies	Light (0–12 g/d), Moderate (12–24 g/d), or Heavy (>24 g/d)		↓	Li XH et al, 2016
	Case Control	250/1012	Nondrinkers, 0.01–4.9, 5–14.9, 15–24.9, and >25 g alcohol/d	2010–2013 Sweden Epidemiological study of risk factors for LADA and type 2 diabetes	↓	Rasouli B et al, 2014
Multiple Sclerosis	Animal Research	19–20/group	Moderate (2.6% alcohol)	65 d	↓	Caslin B, Maguire C et al, 2019
	Case Control	745/1761	Low (<50 g/week for women and <100 g/week for men), moderate (50–112 g/week for women and 100–168 g/week for men), and high (>112 g/week for women and >168 g/week for men)	2005–2011 Epidemiological Investigation of Multiple Sclerosis 2009–2011 Environment in Multiple Sclerosis studies Sweden	Dose-dependent inverse association	Hedstrom AK et al 2014
	Case Control	1717/4685	No consumption, 0 units/week for women (men); low: 1–7 (1–14); moderate: 8–14 (15–21); and high > 14	2009–2014 Danish MS Biobank Denmark	Inverse association	Andersen C et al, 2019
	Cohort	923	Non drinker, <1 drink/week, >1-4 drinks/week, >4 drinks/week	Comprehensive Longitudinal Investigation of Multiple Sclerosis at the Brigham and Women’s Hospital United States	↓	Diaz-Cruz C et al, 2017
	Cross-sectional	429/547,288	Alcohol abuse, alcohol dependence, and alcohol use	1999–2011 English National Health Service hospitals	↑	Pakpoor J et al, 2014
	Cross-sectional	258/238,371	0 g/d, 0.1–4.9 g/d, 5.0–14.9 g/d, 15.0–29.9 g/d, and 30+ g/d	1980–2004 Nurse Health Study 1 1991–2005 Nurse Health Study 2	No effect	Mass J et al, 2013

### Autoimmune diabetes

The beneficial effects of moderate alcohol have been documented in both non-autoimmune type 2 diabetes and in autoimmune type 1 diabetes in adults (LADA)^30–34^ (Table 1). In a study of LADA, there was a 60% risk reduction in patients who consumed 2–7 g/day compared with patients consuming 0.01–2 g/day. This study also noted higher anti‐glutamic acid decarboxylase antibody (GAD Ab) levels and lower C‐peptide in abstainers compared with alcohol consumers, with a more pronounced effect in men. In another LADA study, a 46% risk reduction was noted in men and women consuming greater than 25 g/day.^34^ The effect appeared to be strongest in patients with low anti-GAD Ab levels and was restricted to wine drinkers compared to beer or liquor consumers.^34^ The authors surmised that patients with low anti-GAD Ab levels may be most similar to patients with type 2 diabetes, and it may be polyphenols and hydroxystilbenes in wine that promote anti-oxidative or anti-inflammatory effects of alcohol in autoimmunity.^34^

### Autoimmune thyroid diseases

Similarly to diabetes, moderate alcohol has been demonstrated to be protective in both autoimmune hypothyroidism and hyperthyroidism (Table 1). For example, moderate alcohol was correlated with decreased risk of hypothyroidism and Grave’s disease in a dose-dependent manner compared to controls, regardless of gender or type of alcohol consumed.^10,35^ Several studies also found that moderate alcohol consumption of >10 units/week^36^ or at least 35 g of alcohol per day^37^ was associated with a lower probability of autoimmune thyroid disease and development of positive thyroid peroxidase antibodies.

### Systemic lupus erythematosus

A significant dose-dependent association between moderate alcohol and SLE risk has been identified in multiple case-control, cohort and cross-sectional studies^9,38–41^ (Table 1). In a meta-analysis, protective effects of moderate alcohol were tied to the duration of SLE, with significance seen in patients treated for less than 10 years compared to patients treated for less than 5 years.^40^ Another study concluded that moderate alcohol may lower the chance of ANA-positive patients to progress to SLE.^42^ Smaller case–control SLE studies, which tend to be more prone to recall bias and reverse causation bias, have either not identified an association with alcohol consumption and SLE risk or have detected a slightly higher risk.^43,44^

### Rheumatoid arthritis

Similar to thyroid disease, diabetes and SLE, multiple epidemiological studies and several mechanistic studies support the protective role of light to moderate alcohol in RA in a J- or U-shaped dose-dependent manner^8,15,45–54^ (Table 1). In a meta-analysis study, both men and women had a reduction in RA risk over 10 years, with women experiencing the highest risk reduction.^49^ In other studies, women consuming moderate alcohol reported lower disease activity and higher quality of life compared to men.^48^ However, it has also been documented that alcohol may prevent radiological progression in men and increase radiological progression in women.^53^ A significantly lower Modified Health Assessment Questionnaire scores (suggestive of improved functional status) have also been found in RA patients consuming moderate alcohol compared to nondrinkers.^55^ This effect was stronger in patients who were positive for *HLA-DRB1* shared epitope.^55^ Thus, there is likely to be a beneficial but complex relationship between alcohol, gender and genetic make-up in RA.

### Multiple sclerosis

Likewise, in MS there is also evidence for protective effects of moderate alcohol in decreasing disease risk and/or disease progression (Table 1). Several large population studies have demonstrated a dose-dependent inverse association between alcohol and MS risk in both sexes.^12,56^ Moderate consumption of red wine appears to correlate with a lower Expanded Disability Status Scale score, suggesting improved function, though patients drinking moderate alcohol exhibited an increase in T2 lesion volume on brain MRI.^57^ Conversely, high doses of alcohol may contribute to increased risk of MS, particularly in men.^58,59^

Some studies have noted no association between different doses of alcohol and the risk of developing MS. In these studies, gender may be a variable that may explain alcohol’s effects in MS. For example, in a female Nurses’ Health Study (NHS) I and II, there was no association between different types of alcohol and the risk of MS.^60^ Although this was a large study of >90,000 women between the two NHS studies, the cohort of MS patients of 258 cases was relatively smaller and it is also possible that females may not experience the degree of protective effects of alcohol compared to males. For example, in an animal model of MS, experimental autoimmune encephalomyelitis (EAE), it was recently shown that primarily male mice improved in disease scores on a moderate alcohol diet.^11^

## Alcohol’s pro- and anti-inflammatory effects on the immune system

It is well known that chronic high-dose alcohol consumption can lead to a higher infectious disease burden in alcoholics and more difficulty in clearing pathogens such as *Listeria monocytogenes*, *Mycobacterium tuberculosis* and influenza.^61–63^ However, while chronic high-level alcohol consumption is known to induce systemic inflammation, there is an increasing understanding that alcohol’s effects on the innate and adaptive immune system are dose-dependent.

For example, alcohol has prominent dose-dependent effects on microglia, the innate immune cells of the central nervous system (CNS). In mouse models of acute alcohol abuse, cerebellar microglia display no inflammatory cytokine production following a single moderate-dose alcohol exposure of 3 g/kg and only a transient IL-1β/TNF-α increase following high-dose administration 5 g/kg.^64^ At much higher alcohol doses of up to 10 g/kg/day, microglia display increased activation in association with the production of different inflammatory cytokines, including IL-1β, IL-18, IL-10, interferon-gamma (IFN-γ), transformative growth factor beta (TGF-β) and chemokines, CXCL2, CX3CL1. In turn, these cytokines and chemokines can lead to peripheral lymphocyte translocation across the blood–brain barrier (BBB) and further CNS inflammation.^65–68^

Alcohol also modulates the adaptive immune system in a dose-dependent manner. Chronic moderate alcohol consumption leads to T and B cell activation and proliferation,^69^ while chronic heavy consumption is associated with T and B cell depletion and apoptosis as well as an increase in immunoglobulins.^70^ Additionally, chronic binge alcohol consumption changes T cell phenotypes leading to a decreased percentage of naive T lymphocytes and higher percentages of memory T-cells.^71,72^ Conversely, moderate alcohol consumption has been linked to modulation of T follicular helper (T_FH_) cells.^8^

Cytokines and inflammatory markers are also affected by alcohol in a dose-dependent manner. For instance, C-reactive protein (CRP) and interleukin 6 (IL-6) are elevated in human heavy drinkers but relatively reduced in moderate drinkers compared to nondrinkers.^73^ CRP effects may also be sexually dimorphic, with some studies indicating that alcohol-induced CRP reduction is specific to women,^74^ though other studies suggest that moderate consumption reduces CRP in a U-shaped pattern regardless of sex.^75^

### Pro- and anti-inflammatory dose-dependent alcohol effects on the immune system in rheumatoid arthritis

In RA, a J-shaped association has been noted with CRP levels, with patients consuming 1–7 drinks/week had the lowest CRP levels.^51^ RA patients consuming moderate alcohol display a U-shaped association with IL-6 levels prior to symptom development and an inverse relationship between alcohol consumption and soluble tumor necrosis factor receptor 2 (TNFR2) levels.^76^ As alcohol can contribute to liver damage, a study evaluating the relationship between alcohol consumption and liver inflammation reported that >21 units per week correlated with transaminitis, while <14 units per week did not.^48^ Moderate alcohol consumption has also been associated with 50% reduction in RA risk in patients positive for anticitrullinated protein antibodies (ACPA), and a 30% disease risk reduction in ACPA-negative RA in an inverse dose–response relationship.^47^

Dose-dependent effects of alcohol on the immune system are also noted in RA mouse models. In a model of collagen-induced arthritis (CIA), mice on a moderate alcohol diet experienced a 40% lower incidence of CIA and >50% decrease in radiological disease severity compared to non-alcohol controls.^8^ Alcohol-consuming mice also had lower levels of IL-21 and IL-17A, neutrophils, monocytes, plasma B cells and IgG levels.^8^ The authors also found that both alcohol and acetate affected the functional state of T follicular helper (T_FH_) cells in vitro and in vivo, leading to suppression of IL-21 secretion.^8^ These findings are intriguing as T_FH_ cells are often found in synovial joints in RA patients and are also important mediators of gut immunity, suggesting a possible link between immune processes in the gut and RA. In another CIA study, moderate alcohol (10% ethanol in water) delayed the onset and ameliorated the progression of CIA via an increase in endogenous testosterone, inhibition of nuclear factor B activation and down-regulation of leukocyte migration.^77^

## Alcohol’s pro- and anti-inflammatory effects on the gut

The gut microbiome, composed of trillions of microorganisms, is increasingly considered to be one of the critical environmental factors in modulating the immune system and risk of autoimmune diseases. For example, fecal transplantation of gut bacteria from patients with SLE and MS to animal models can reproduce disease symptoms in animals.^78,79^ Conversely, supplementation of a dysbiotic microbiome with diverse commensal species through fecal transplant has therapeutic benefits in IBS and MS.^80–82^ Similarly, in animal studies, antibiotic ablation of the gut flora is sufficient to prevent EAE onset entirely, while the introduction of strains of Erysipelotrichaceae and *Lactobacillus reuteri* exacerbates EAE symptoms and increases autoreactive T-cells.^83^ In an RA model in germ-free mice, the introduction of segmented filamentous bacteria (SFB) was sufficient for the development of arthritis via Th17 response.^84^ Likewise, in non-germ-free RA mice, oral gavage of SFB drove the differentiation and migration of T_FH_ cells systemically, leading to autoantibody generation and arthritis exacerbation.^85^

As alcohol is largely metabolized within the GI tract, it is a prime factor to impact gut microbiome composition, gut immune system and downstream systemic immune communications with other organs. In the following section, we will focus on alcohol’s effects on the gut, gut immune system and gut metabolism of fatty acids and how these effects may translate into pro-inflammatory vs protective effects in autoimmune diseases.

### Pro-inflammatory effects of high-dose alcohol on the gastrointestinal tract, gut microbiome, gut metabolites and nutrients

Microbiome transfer from high-dose alcohol-fed mice to alcohol-naïve germ-free mice has been shown to induce intestinal inflammation in the recipient mice.^86^ Potential explanations for how high-dose alcohol may induce gut inflammation include alteration of the gut microbiome (dysbiosis) in alcohol-metabolizing species, bacterial cytotoxicity, changes to enteric peristalsis and hepatotoxicity (liver steatosis and cirrhosis).^87–90^ Microbiome dysbiosis can lead to impaired intestinal permeability and promote inflammation via systemic translocation of gut bacterial endotoxin, LPS, activation of TLR and nuclear factor-κB (NF-kB) on immune cells and induction of inflammatory iNOS^17,19,20.^ In turn, hepatotoxicity interferes with the liver’s ability to detoxify substances, which results in systemic accumulation of alcohol’s toxic metabolite, acetaldehyde.^91^ In addition, LPS-mediated activation of liver-resident macrophages, Kupffer cells, further contributes to pro-inflammatory cytokine release and propagation of systemic inflammation.^92^

There are also specific microbiota changes that have been described in animal and human high-dose alcohol studies (Table 2). In human alcohol use disorder (AUD) studies, dysbiosis has been characterized by lower Bacteroidetes,^89,93^ lower *Akkermansia muciniphila*,^94,121,95^ and higher Proteobacteria.^89^ In animal models of high-dose alcohol consumption, alcohol-consuming animals have reduced bacterial diversity along with lower Bacteroidetes, elevated Proteobacter, elevated Actinobacter,^94^ reduction in Firmicutes and elevation in Bacteroidetes.^96^ However, not all studies have noted a reduction in Firmicutes in response to high-dose alcohol. For example, voluntary self-administration of chronic high-dose alcohol in macaques resulted in reduced Bacteroidetes, elevated Firmicutes and a complete absence of *Akkermansia muciniphila* during the drinking period, while abstinence from drinking restored baseline bacterial species.^90^

**Table 2.** Alcohol induced gut microbiota alterations by dose and alcohol diet duration

**Alcohol Model**	**Alcohol Model Details**	**Diet Duration**	**Subject Model**	**Microbiota**	**Reference**
Chronic(Alcohol Liver Disease)	National Institute on Alcohol Abuse and Alcoholism, criteria for alcoholism (Men: >14 drinks/wk or >4 drinks/occasion Women: >7 drinks/wk or >3 drinks/occasion)	Alcohol consumption ≥10 yrs	Human	↑Proteobacteria↑Gammaproteobacteria↑Bacilli↓Clostridia↓Bacteriodetes↓Verrucomicrobiae	Mutlu et al., 2012
Chronic (Alcohol Dependence (AD))	Diagnostic and Statistical Manual of Mental Disorders IV criteria for AD	19 days of rehab/alcohol abstinence in patients with AD	Human	↑Ruminococcaceae↑Bifidobacterium↑Lactobacillus↓Erysipelotrichaceae↓Holdemania	Leclercq et al., 2014
Chronic (Alcohol Use Disorder (AUD))	Less heavy drinkers (<10 drinks/d) vs high heavy drinkers (≥10 drinks/d)	4 weeks of abstinence in patients with AUD	Human	Less Heavy Drinkers↑ Erysipelotrichaceae↑ Lachnospiraceae	Ames et al., 2020
Chronic (Alcohol Dependence Syndrome (ADS))	ICD-10 definition of alcohol dependence syndrome +/- liver cirrhosis	ADS: ≥8 years	Human	*↑ Klebsiella↑ Lactobacillus↑ Bifidobacterium↓ Prevotella,↓ Faecalibacterium prausnitzii↓ Acidaminococcus↓ Clostridiales*	Dubinkina et al., 2017
Chronic	0.8-2.2 g/kg	5 years	Macaque	↑Firmicutes: Streptococcaceae↑ Firmicutes : Bacteriodes ratio	Zhang et al., 2019
Chronic	2.6% Lieber-DeCarli diet	65 days	Mouse	*↑ Turicibacter↑ Akkermansia↑ Prevotella↑ Clostridium↑ Verrucomirobia : Firmicute*	Caslin, Maguire et al., 2019
Chronic	Vapor chamber 175 ± 25 mg/dL	4 weeks	Mouse	↑Rikenellaceae↑Alistipes↓Clostridium lV↓Clostridium XiVb ↓Dorea↓Coprococcus ↓Propionibacterium	Peterson et al., 2017

In addition to changes in microbiota, high-dose alcohol can also decrease immunomodulatory gut metabolites such as aryl hydrocarbon receptor (AhR).^97^ AhR is a transcriptional factor expressed in immune cells and is known to impact T cell differentiation, effector and regulatory T cell functions. AhR aberrant expression has also been linked to autoimmune dysregulation.^98,99^ As AhR ligand administration is beneficial in autoimmune diseases, lower levels of AhR due to chronic high-dose alcohol may potentially contribute to autoimmune disease exacerbation.

Lastly, chronic high-dose alcohol intake can cause malabsorption and nutrient deficiencies.^100^ In turn, nutrient deficiencies for vitamins such as thiamine, cyanocobalamin and vitamin D can exacerbate different autoimmune conditions.^101^

### Anti-inflammatory effects of low-to-moderate alcohol on the gut microbiome, gut metabolites and fatty acids

An important way in which alcohol may beneficially impact autoimmune inflammation is via its effects on fatty acid metabolism in the gut. While at high doses alcohol is known to lead to fatty acid dysregulation and development of fatty liver disease,^102–104^ at lower doses, alcohol may contribute to the generation of gut-derived anti-inflammatory fatty acids, such as short-chain fatty acids (SCFAs) and polyunsaturated fatty acids (PUFAs)^103,104^ (Figure 1).

SCFAs, including formate, acetate, propionate and butyrate, are a class of carboxylic acids produced primarily through microbial fermentation of dietary fiber.^106^ Importantly, SCFAs are known to have important anti-microbial and anti-inflammatory properties and to reduce inflammation in autoimmune diseases.^107,108^ Oral supplementation of SCFA cocktail has been shown to be sufficient to reduce autoreactive Th1 and Th17 cell activity and to dampen disease severity in animal models of autoimmune colitis and EAE.^109,110^

There are potentially two ways in which low-to-moderate alcohol consumption can modulate SCFA production. First, low-to-moderate alcohol can alter SCFA-producing microbial communities in the gut, such as *Akkermansia muciniphila*.^111^ Caslin, Maguire et al. showed that moderate alcohol consumption (2.6% ABV Lieber-DeCarli diet) increased levels of *Akkermansia muciniphila* in the gut in association with reducing disease severity in EAE.^11^ Similarly, Lee et al. found that short-term alcohol consumption (5 days of 0.8 g/kg intragastric) elevated *Akkermansia muciniphila* levels in mice, an elevation not observed in groups consuming fermented rice liquor (FRL) of equivalent alcoholic strength.^112^ In addition, alcohol itself is metabolized into the SCFA, acetate,^113^ and animals fed a Lieber-DeCarli diet for 8 weeks show elevated levels of acetic acid compared to controls.^114^ Thus, low-to-moderate alcohol could impact SCFA balance both by influencing SCFA producing bacteria and via acetate production (Figure 1).

Another potential mechanism of low-to-moderate alcohol’s protection in autoimmune diseases may rely on alcohol’s important role in the metabolism of essential PUFAs, such as docosahexaenoic acid (DHA) and eicosapentaenoic acid (EPA).^103,105^ These PUFAs can reduce reactive oxygen species formation and act as anti-inflammatory molecules. Low-to-moderate dose alcohol has been shown to increase PUFA production,^115^ while at high alcohol doses, PUFA concentration decreases due to increased fatty acid catabolism.^103^ Of note, PUFAs and PUFA derivatives, such as resolvins, lipoxins and protection, have been linked to the mitigation of autoimmune diseases.^116,117^ In addition, an increase in PUFAs has also been shown to be cardioprotective in multiple studies.^118–120^ As cardiovascular health is becoming an important factor in autoimmune disease outcomes, it is possible that this may be another protective mechanism mediated by low-to-moderate alcohol (Figure 1).

### Protective effects of low-moderate alcohol on the gut microbiome in models of multiple sclerosis

In an animal model of MS, EAE, Caslin, Maguire et al. administered a 2.6% moderate alcohol or isocaloric diet to both male and female C57BL/6 J mice and observed that males on the diet exhibited a greater long-term disease remission while females initially experienced remission and a subsequent disease exacerbation.^11^ In this study, a moderate alcohol diet resulted in sex-specific gut microbiota alterations in individual immunoregulatory taxa, such as *Turicibacter, Akkermansia* and *Prevotella*, and also led to significant enrichment in beneficial Clostridial and Firmicute networks of bacteria.^11^ Another study evaluating a moderate alcohol model of 10% (vol/vol) in EAE also documented EAE amelioration in alcohol-consuming C57BL/6 J mice and implicated gut-related T_FH_ cells mechanistically.^8^

## Conclusions and future directions

Overall, current evidence points to a dose-dependent association between alcohol and disease severity in multiple autoimmune diseases, including autoimmune thyroid disease, diabetes, SLE, RA and MS. At low-to-moderate doses, alcohol appears to have protective effects, while at higher consumption patterns, alcohol can be addictive and can contribute to detrimental symptomatic effects on the host and worse autoimmune disease outcomes.

Though the exact mechanism by which low-to-moderate alcohol mediates autoimmune disease amelioration remains to be fully understood, emerging mechanistic studies suggest that low-to-moderate alcohol likely has both a systemic immunomodulatory role, such as shifting the balance of anti-inflammatory innate and adaptive immune cells and cytokines/chemokines, as well as a role in sculpting the composition of the gut microbiome and their fatty acid metabolites, such as SCFAs and PUFAs.

Future prospective patient studies that account for sex, age, cultural and socioeconomic background, alcohol type, timing of administration and mechanistic animal model studies on the gut microbiome and the immune system will be critical to better understand alcohol’s role in autoimmune diseases. In turn, this knowledge will help guide the creation of specific clinical recommendations on alcohol consumption in patients with autoimmune diseases as well as help identify protective immune and gut-derived biomarkers that could be used in the treatment of autoimmune diseases independently of alcohol.
